# Resveratrol ameliorates diet-induced dysregulation of lipid metabolism in zebrafish (*Danio rerio*)

**DOI:** 10.1371/journal.pone.0180865

**Published:** 2017-07-07

**Authors:** Gai Ran, Li Ying, Lin Li, Qiaoqiao Yan, Weijie Yi, Chenjiang Ying, Hongmei Wu, Xiaolei Ye

**Affiliations:** 1 Department of Preventive Medicine, School of Public Health and Management, Wenzhou Medical University, Wenzhou, China; 2 School of Stomatology, Wenzhou Medical University, Wenzhou, China; 3 Department of Nutrition and Food Hygiene, School of Public Health, Tongji Medical College, Huazhong University of Science and Technology, Wuhan, China; Bose Institute, INDIA

## Abstract

Defective lipid metabolism is associated with increased risk of various chronic diseases, such as obesity, cardiovascular diseases, and diabetes. Resveratrol (RSV), a natural polyphenol, has been shown the potential of ameliorating disregulations of lipid metabolism. The objective of this study was to investigate the effects of feed intake and RSV on lipid metabolism in zebrafish (*Danio rerio*). The adult males were randomly allocated to 6 groups: control (Con, 8 mg cysts/fish/day), control with 20 μmol/L RSV (Con+RSV), calorie restriction (CR, 5 mg cysts/fish/day), calorie restriction with RSV (CR+RSV), overfeed (OF, 60 mg cysts/fish/day), and overfeed with RSV (OF+RSV) groups. The treatment period was 8 weeks. Results showed that CR reduced body length, body weight, and condition factor of zebrafish. CR reduced levels of plasma triglyceride (TG) and induced protein expression of phosphorylated AMP-activated protein kinase-α (pAMPKα), silent information regulator 2 homolog 1 (Sirt1), and peroxisome proliferator activated receptor gamma coactivator-1α (PGC1α). RSV attenuated CR-induced pAMPKα/AMPKαincreases. RSV increased levels of Sirt1 protein in the OF zebrafish, and decreased OF-induced increase in peroxisome proliferator-activated receptor-γ (PPARγ) protein level. Additionally, RSV down-regulated caveolin-1 and up-regulated microtubule-associated protein 1 light chain 3 -II (LC3-II) protein levels in OF zebrafish. In conclusion, these results suggest that 1) CR reduces plasma TG level through activation of the AMPKα-Sirt1- PGC1α pathway; 2) under different dietary stress conditions RSV might regulate AMPK phosphorylation bi-directionally; 3) RSV might regulate lipid metabolism through the AMPKα-Sirt1-PPARγ pathway in OF zebrafish.

## Introduction

Differences in calorie intake contribute to the formation of various somatotypes that affect optimal health. High-fat diet enhances an excess of energy intake, resulting in excessive fat accumulation and an increased risk of acquiring a number of chronic diseases, such as obesity, cardiovascular diseases, and diabetes [1]. Conversely, calorie restriction (CR), a 30%–50% reduction in dietary intake relative to *ad libitum*, has been proven to be effective in extending lifespan and protecting against obesity, cardiovascular disease, and diabetes in various animal models, such as fish, rats and mice [2, 3].

Resveratrol (trans-3,5,4’-trihydroxystilbene, RSV) is a natural polyphenolic compound found in various plants, such as grapes, blueberries, raspberries, mulberries, and peanuts, as a potential health-promoting compound. RSV has been shown to exert anti-inflammatory, antioxidant, anti-obesity, cardioprotective effects, etc [4, 5]. In high-fat-diet-induced obese mice, RSV exerts anti-obesity effects through alterations in lipid metabolism-related gene expression [6], decreases in preadipocyte differentiation and lipogenesis, and increases in lipolysis and fatty acid β-oxidation [7, 8]. Meanwhile, RSV is known to induce autophagy and regulate mobilization and degradation of lipid droplets [9, 10].

Adenosine monophosphate-activated protein kinase (AMPK) is considered to be involved in cellular energy homeostasis. The AMPK pathway is activated through auto-phosphorylation of AMPK (pAMPK) on Thr-172. Activation of silent information regulator 2 homolog 1 (Sirt1) by pAMPK subsequently affects peroxisome proliferator-activated receptor gamma coactivator-1-alpha (PGC1α) through deacetylation [11, 12]. Sirt1 and PGC1α are vital in improvement of metabolic fitness. Up-regulation of Sirt1 triggers lipolysis and loss of fat [13]. Furthermore, PGC1α has been extensively described as a master regulator of fatty acid oxidation and gluconeogenesis [12]. Besides the pathways mentioned above, either directly or through Sirt1, pAMPK suppresses expression of peroxisome proliferator-activated receptor gamma (PPARγ) [14, 15], which promotes adipocyte differentiation and stimulates fat storage in adipocytes [16].

The AMPK-Sirt1 pathway may regulate autophagy. During autophagy, the conversion of microtubule-associated protein 1 light chain 3 (LC3) from LC3-I (free form) to LC3-II (conjugated form) represents a key step in autophagosome formation [17]. Thereby, LC3-II is considered as an indicator of autophagy induction. Through Sirt1, pAMPK induces LC3-II expression and subsequently autophagy [14]. *In vitro* studies show that RSV increases LC3 expression *via* the AMPK-Sirt1 pathway [11]; however, *in vivo* studies are relatively limited.

Caveolin-1 (Cav-1), a membrane scaffolding protein, is the main structural component of caveolae and plays a crucial role in autophagy-mediated maintenance of cellular cholesterol homeostasis and lipid transportation [18]. In adult rats fed with high-fat diets, RSV has been shown to down-regulate the expression of Cav-1 and induce autophagy in adipocytes, which are associated with the maintenance of the homeostasis in lipid metabolism [19, 20].

Thus, we hypothesized that restricted or excessive calorie intake are related to dysregulation of lipid metabolism, and RSV has the potential in maintenance of *in vivo* homeostasis of lipid metabolism in zebrafish. Zebrafish models are ideal and convenient for studies on human diseases as they are relatively easy to maintain and genetically manipulate, cost-effective, high in fecundity, and similar to humans. Importantly, the model of overfeed zebrafish shares common pathways with obese mammals in pathophysiological aspects. Firstly, digestive organs, adipose tissues, and skeletal muscle of zebrafish are physically similar to the human counterparts. Secondly, the absorption, transportation, and metabolism of fatty acid and lipid in zebrafish are very similar to human. Thirdly, neural and endocrine signals regulating feed intake are conserved in zebrafish and mammals [21–24]. Therefore, zebrafish is used to determine the effects of RSV on defective lipid metabolism induced by diet in this study.

Here, we investigated effects of various feed intake and RSV on lipid metabolism in zebrafish, and explored potential regulatory pathways. These findings provide new insight for preventive and therapeutic measures of metabolic disorders induced by overfeeding.

## Materials and methods

### Ethics statement

The research protocol was approved by the Institutional Animal Care and Use Committee (IACUC) at the Wenzhou Medical University, and all the experiments were performed as approved. All dissections were conducted on ice.

### Reagents

Resveratrol was purchased from Nanjing Spring & Autumn Biological Engineering Co., Ltd. (Nanjing, China). Anti-pAMPKα (Thr172) (40H9) and anti-Cav-1 (D46G3) were from Cell Signaling Technology. Anti-LC3 (NB600-1384) was from NOVUS. Anti-PPARγ (H-100) was from Santa Cruz Biotechnology. Anti-PGC1α (ab54481) was from ABcam. Anti-AMPKα (10929-2-AP), anti-Sirt1 (13161-1-AP) and anti-GAPDH (60004-1-lg) were from Proteintech (Wuhan, China).

### Zebrafish strains and treatments

Wild-type (AB strain) zebrafish *(Danio rerio)* were obtained from Sinnhuber Aquatic Research Laboratory of Oregon State University (Corvallis, OR, USA) and maintained at Zhejiang Provincial Key Lab for Technology and Application of Model Organisms. Fish were kept at 28°C with a 14 h: 10 h dark: light cycle (lights on at 8:00 AM) in a recirculation system. Zebrafish embryos were obtained from spawning of adult zebrafish with the male and female ratio of 1 to 1. Embryos were collected within 1 h after spawning and rinsed with embryo medium. Fertilized embryos were inspected and staged. When zebrafish embryos were hatched and grew up to adult fish after 4 months, the male and female zebrafish were separated according to the morphological characteristics.

Adult male zebrafish were randomly allocated to 6 groups: control (Con), control with RSV (Con+RSV), calorie restriction (CR), calorie restriction with RSV (CR+RSV), overfeed (OF), and overfeed with RSV (OF+RSV) groups. Fish were fed with freshly hatched live artemia (Huizhong Fisheries Co., Ltd., Shandong, China). They were 8 mg cysts/fish/day for Con groups (once a day), 5 mg cysts/fish/day for CR groups (once a day), and 60 mg cysts/fish/day (three times a day) for OF groups. For RSV treatments, fish were exposed to 20 μmol/L RSV. Forty one mg of RSV was dissolved in 0.9 mL of DMSO then diluted with 9 L of distilled water in tank, obtaining 20 μmol RSV/L water solution. Fish were transferred to the tanks containing 20 μmol/L RSV from 20:00 to 08:00 every day. The treatment duration was 8 weeks.

### Body length, body weight, and Fulton’s condition factor

At the end of the treatment, zebrafish were fasted for 24 h. Then, body length (cm), from the anterior-most region of the mouth to the tail end, and body weight (g) were determined. The Fulton’s condition factor was calculated according to the following formula: condition factor = (Weight/Length^**3**^) ×100.

### Blood glucose, plasma triglyceride (TG), plasma total cholesterol (TC)

After 8 weeks of feeding, adult fish from each group were fasted for 24 h prior to blood collection. Zebrafish were blotted with filter paper and then the blood was collected immediately. Blood glucose was detected by the Johnson ONETOUCH^**®**^ Ultra Easy (Johnson & Johnson Co, USA). Total triglyceride and cholesterol levels were analyzed by plasma lipid kits (Applygen Technologies Inc., Beijing, China).

### Hematoxylin-eosin staining

Fresh livers were fixed in neutral buffered 4% paraformaldehyde (PFA) for 24 h at room temperature. Then, the samples were dehydrated, infiltrated, embedded in paraffin, sliced into 4-μm-thick by pathologic microtome (RM2016, Leica), and then mounted onto glass slides and dried at 60°C. Then, tissue sections were stained by hematoxylin and eosin (H&E) and observed at 400× magnifications with optical microscope (Nikon Eclipse CI, Japan).

### RNA isolation and quantitative real-time PCR analysis

Total RNA was extracted from muscle using RNAsimple Total RNA Kit (TIANGEN Biotech, Beijing, China) according to manufacturer’s instructions. Extracted RNA was quantified, and a total of 500 ng RNA was applied to synthesize cDNA by using PrimeScript RT reagent kit (TaKaRa, Japan). Primers were synthesized by Beijing Genomics Institute (Shenzhen, China). They included: AMPK (forward: 5’-ATCATAGACAACCGCCGCATTA-3’, reverse: 5’-TTGGCTCGCCGTACACCA-3’), Sirt-1 (forward: 5’-CCCTGATCTTCTTCGGGACG-3’, reverse: 5’-GAGGAAGCACCGTTTCAGGA-3’), PPARγ (forward: 5’-TCTCCGCTGATATGGTGGAC-3’, reverse: 5’-GTCGATGCCTGATATGCTGC-3’), PGC1α (forward: 5’-TCAATACCCAGGTGGCAAGG-3’, reverse: 5’-TTTGATGCAAGAAGTGCGGTG-3’), and GADPH (forward: 5’-ACAGCAACACAGAAGACCGT-3’, reverse: 5’-GGCAGGTTTCTCAAGACGGA-3’).

The quantitative real-time polymerase chain reaction (qPCR) was performed using FastStart Essential DNA Green Master (Roche, Indianapolis, IN, USA) on CFX96 Touch^**TM**^ real-time PCR detection system (Bio-Rad Laboratories, Hercules, CA, USA). The amplification condition is: 40 cycles of 95°C for 10 s, 60°C for 30 s, and 72°C for 20 s. The mRNA levels were calculated using the 2^**-ΔΔCt**^ method. GAPDH was used as an internal control for normalization.

### Co-immunoprecipitation and western blotting analysis

Muscle was dissected and immediately frozen in -80°C for later analysis. Co-immunoprecipitation (Co-IP) was performed as previously described [25]. Total protein was extracted from the muscle using RIPA lysis buffer, and quantified by using BIO-RAD DC Protein Assay Reagent (Bio-Rad, Hercules, CA, USA) according to the manufacturer's instruction. Equal amounts of protein were mixed with SDS sample buffer and incubated for 5 min at 98°C before loading. Proteins were separated by sodium dodecyl sulfate-polyacrylamide gel electrophoresis (SDS-PAGE) and transferred to PVDF membranes according to the method of Amersham Biosciences. Each membrane was incubated with primary antibodies overnight at 4°C. Membranes were then washed and incubated with secondary antibody for 1 h at room temperature. The immunoblots were visualized with an ECL detection system (Syngen, Cambridge, UK) and analyzed by software Chemidoc-Quantity-One (Bio-Rad Laboratories).

### Statistical analyses

Data were presented as mean ± SD (Standard Deviation). Data processing and statistical analysis were performed by using SPSS 20.0 (SPSS Inc. Chicago, IL, USA). A 3×2 factorial analysis of variance was performed to determine the main effects of feed intake and RSV, and the interaction. When there are interactions between feed intake and RSV, simple effects analysis was performed with The EMMEANS (estimated marginal means) subcommand. Significant differences between groups were determined by using Fisher’s least significant difference method of post hoc comparisons. A value of *P* <0.05 was considered statistically significant. All figures were processed with SigmaPlot 12.3.

## Results

### Effects of feed intake and RSV on body length, body weight and Fulton’s condition factor

There were significant differences in body length, body weight and Fulton’s condition factor between different feed intake groups. Compared with the Con group, body length and body weight in CR group were decreased by 4.6% and 20.6%, respectively, and those in OF group were increased by 10.4% and 54.2%, respectively. Compared with the Con group, the Fulton’s condition factor in CR group was decreased by 8.5%, and the Fulton’s condition factor in OF group was increased by 13.9%. Effects of RSV treatment were not statistically significant (Table 1).

**Table 1:** Effects of resveratrol on body length, body weight and condition factor in different diet zebrafish.

Feed	Body length(cm)				Body weight (g)				Condition factor(100g/cm^3^)			
	RSV-		RSV+		RSV-		RSV+		RSV-		RSV+	
	n	Mean ± SD	n	Mean ± SD	n	Mean ± SD	n	Mean ± SD	n	Mean ± SD	n	Mean ± SD
Con	97	3.26±0.15^a^	58	3.32±0.20^A^*	97	0.277±0.033^a^	58	0.280±0.032^A^	97	0.803±0.083^a^	58	0.773±0.107^A^
CR	71	3.11±0.11^b^	121	3.10±0.15^B^	71	0.220±0.023^b^	121	0.217±0.027^B^	71	0.735±0.059^b^	121	0.735±0.084^B^
OF	96	3.60±0.17^c^	101	3.59±0.18^C^	96	0.427±0.054^c^	101	0.425±0.061^C^	96	0.915±0.108^c^	101	0.921±0.112^C^
*F*_*Feed*_			451.141				1228.201				188.996	
*F*_*RSV*_			0.619				0.054				0.975	
*F*_*Feed×RSV*_			2.752				0.207				1.707	
*P*_*Feed*_			<0.001				<0.001				<0.001	
*P*_*RSV*_			0.432				0.817				0.324	
*P*_*Feed×RSV*_			0.065				0.813				0.182	

Letters (a, b, c) indicated the multiple comparison results among various feed intake groups without RSV. Capital letters (A, B, C) indicated the multiple comparison results among various feed intake groups with RSV. Same letters indicated no significant difference, different letters indicated significant differences in statistics.

* indicated the significant difference between groups without RSV and with RSV. For those there was no statistically significant difference between groups, the letters were not shown. Significance level was 0.05.

### Effects of feed intake and RSV on blood glucose, plasma triglyceride and plasma total cholesterol levels

Feed intake did affect fasting blood glucose level. Fasting blood glucose level of OF group was 53.4% higher than Con group, and 35.1% higher than CR group. RSV treatment did not change fasting blood glucose level (Table 2).

**Table 2:** Effects of resveratrol on blood glucose, plasma total cholesterol and triglyceride in different diet zebrafish.

Feed	Blood glucose (mmol/L)				TG (mmol/L)				TC (mmol/L)			
	RSV-		RSV+		RSV-		RSV+		RSV-		RSV+	
	n	Mean ± SD	n	Mean ± SD	n	Mean ± SD	n	Mean ± SD	n	Mean ± SD	n	Mean ± SD
Con	11	6.12±1.22^a^	11	7.43±1.63^A^	5	7.93±1.04^a^	5	6.16±1.52^A^*	5	4.37±0.45^ab^	5	4.23±0.68^AB^
CR	8	6.95±1.19^a^	17	5.81±1.48^B^	5	5.16±1.39^b^	5	3.06±0.56^B^*	5	4.25±0.29^a^	5	3.64±0.52^A^
OF	11	9.39±3.06^b^	14	8.54±1.96^A^	5	6.70±0.83^a^	5	7.20±0.32^A^	5	4.94±0.57^b^	5	4.28±0.28^B^*
*F*_*Feed*_			13.169				25.929				4.672	
*F*_*RSV*_			0.250				8.748				6.884	
*F*_*Feed×RSV*_			2.854				4.664				0.879	
*P*_*Feed*_			<0.001				<0.001				0.019	
*P*_*RSV*_			0.619				0.007				0.015	
*P*_*Feed×RSV*_			0.065				0.019				0.428	

Letters (a, b, c) indicated the multiple comparison results among various feed intake groups without RSV. Capital letters (A, B, C) indicated the multiple comparison results among various feed intake groups with RSV. Same letters indicated no significant difference, different letters indicated significant differences in statistics.

* indicated the significant difference between groups without RSV and with RSV. For those there was no statistically significant difference between groups, the letters were not shown. Significance level was 0.05.

Both feed intake and RSV affected plasma TG levels, and the effects of feed intake and RSV interacted. Plasma TG level was decreased by 34.9% in CR compared to Con group. TG level in Con+RSV group was reduced by 22.3% (*P* <0.05) compared to Con group, and TG level in CR+RSV group was reduced by 40.7% (*P* <0.01) compared to CR group.

Both feed intake and RSV affected plasma TC level. Plasma TC level in OF group was enhanced by 16.2% compared to CR group. RSV treatment reduced TC level by 13.4% in OF zebrafish (Table 2).

### Morphological changes of liver tissues

**Fig 1:**
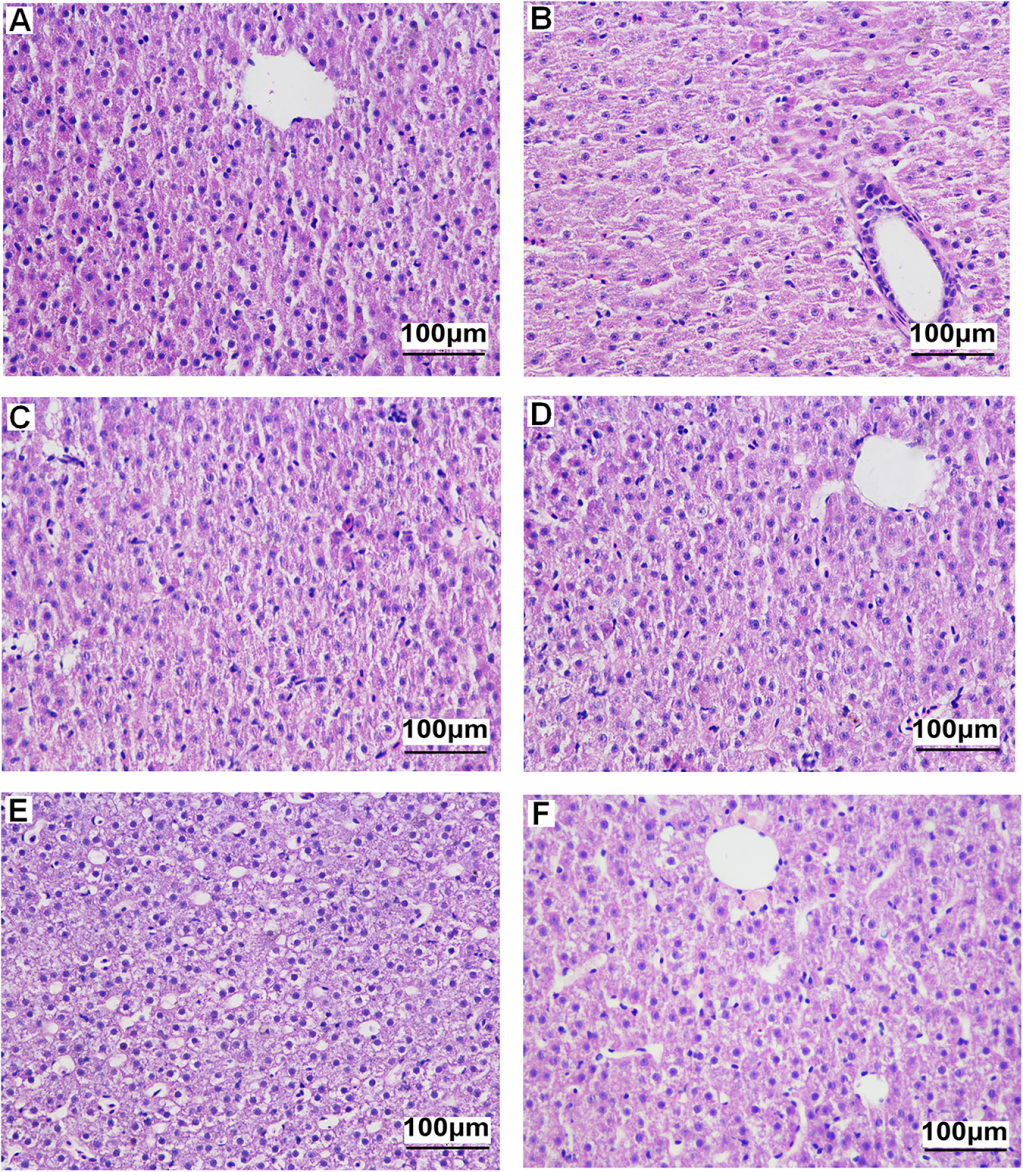
Histopathological observation of liver tissue in zebrafish (×400 magnification, HE staining). A. Con, B. Con+RSV, C. CR, D. CR+RSV, E. OF, F. OF+RSV.

In OF zebrafish, fatty infiltration, hepatocyte ballooning, and irregular arrangement and rupture of hepatocyte were observed. Administration of RSV relieved the destroyed hepatic structure in OF zebrafish (Fig 1).

### Effects of feed intake and RSV on mRNA and protein levels in muscle of zebrafish

**Fig 2:**
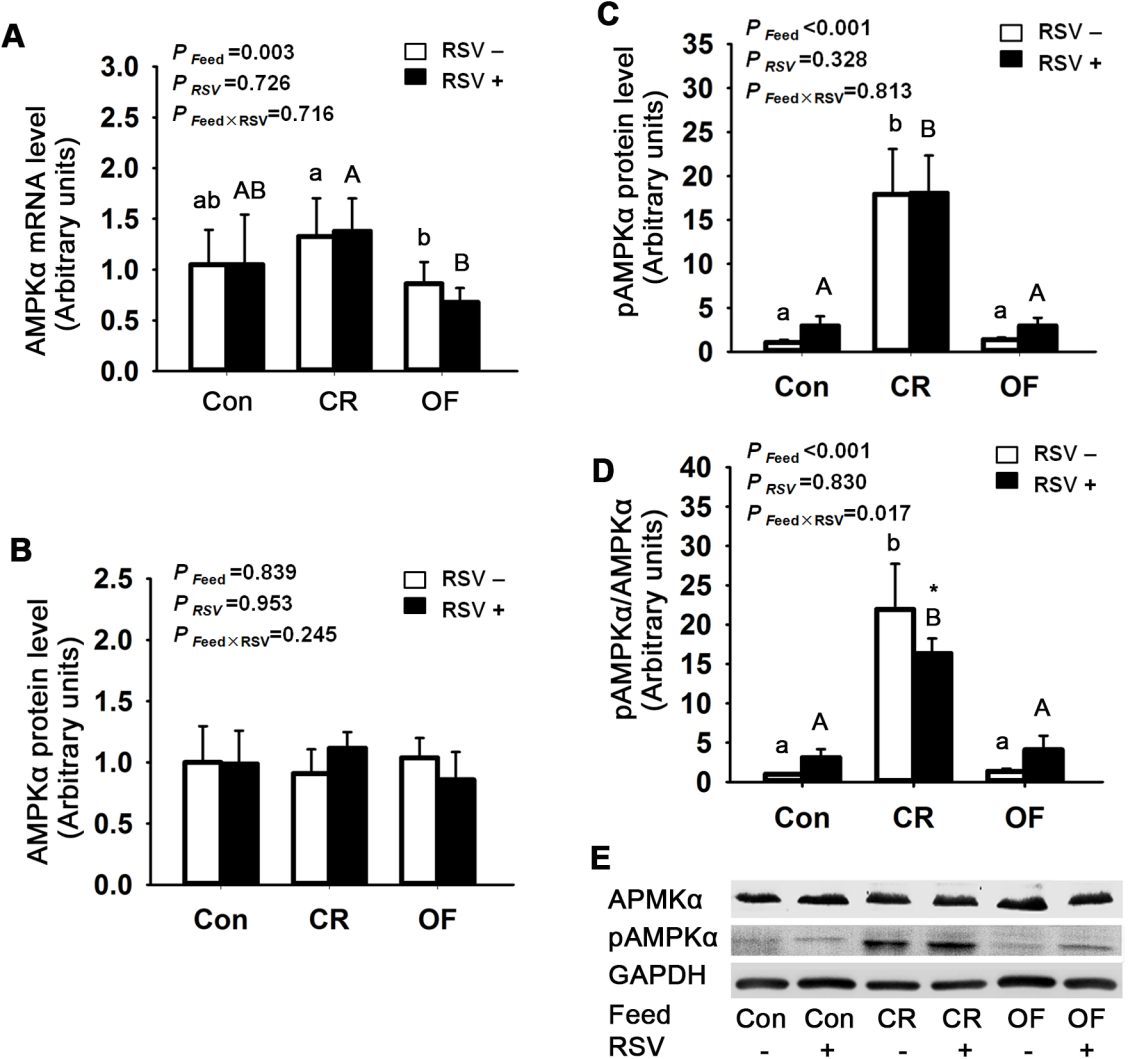
Effects of feed intake and RSV on AMPKα and pAMPKα levels in zebrafish muscle. A. AMPK**α** mRNA levels, B. AMPK**α** protein levels, C. pAMPK**α** protein levels, D. Ratio of AMPK**α** protein level to pAMPK**α** protein level, E. Immunoblots of AMPK**α** and pAMPK**α**. The data were presented as Mean ± SD. Letters (a, b, c) indicated the multiple comparison results among various feed intake groups without RSV. Capital letters (A, B, C) indicated the multiple comparison results among various feed intake groups with RSV. Same letters indicated no significant difference, different letters indicated significant differences in statistics. * indicated the significant difference between groups without RSV and with RSV. For those there was no statistically significant difference between groups, the letters were not shown. Significance level was 0.05.

In muscle, there was no observed change in the expressions of AMPKα mRNA and protein. However, there were significant changes in pAMPKα protein level and pAMPKα/AMPKα ratio between different feed intake groups (*P* <0.001). Compared with Con group, pAMPKα protein level was increased by 15.7-fold (*P* <0.001) in CR group. The pAMPKα/AMPKα ratio in CR+RSV group was reduced by 25.4% (*P* <0.05) compared to CR group. The pAMPKα/AMPKα ratio in OF+RSV zebrafish was 2.0-fold higher than the OF zebrafish. The interaction effect of pAMPKα/AMPKα ratio was observed between feed intake and RSV (Fig 2).

**Fig 3:**
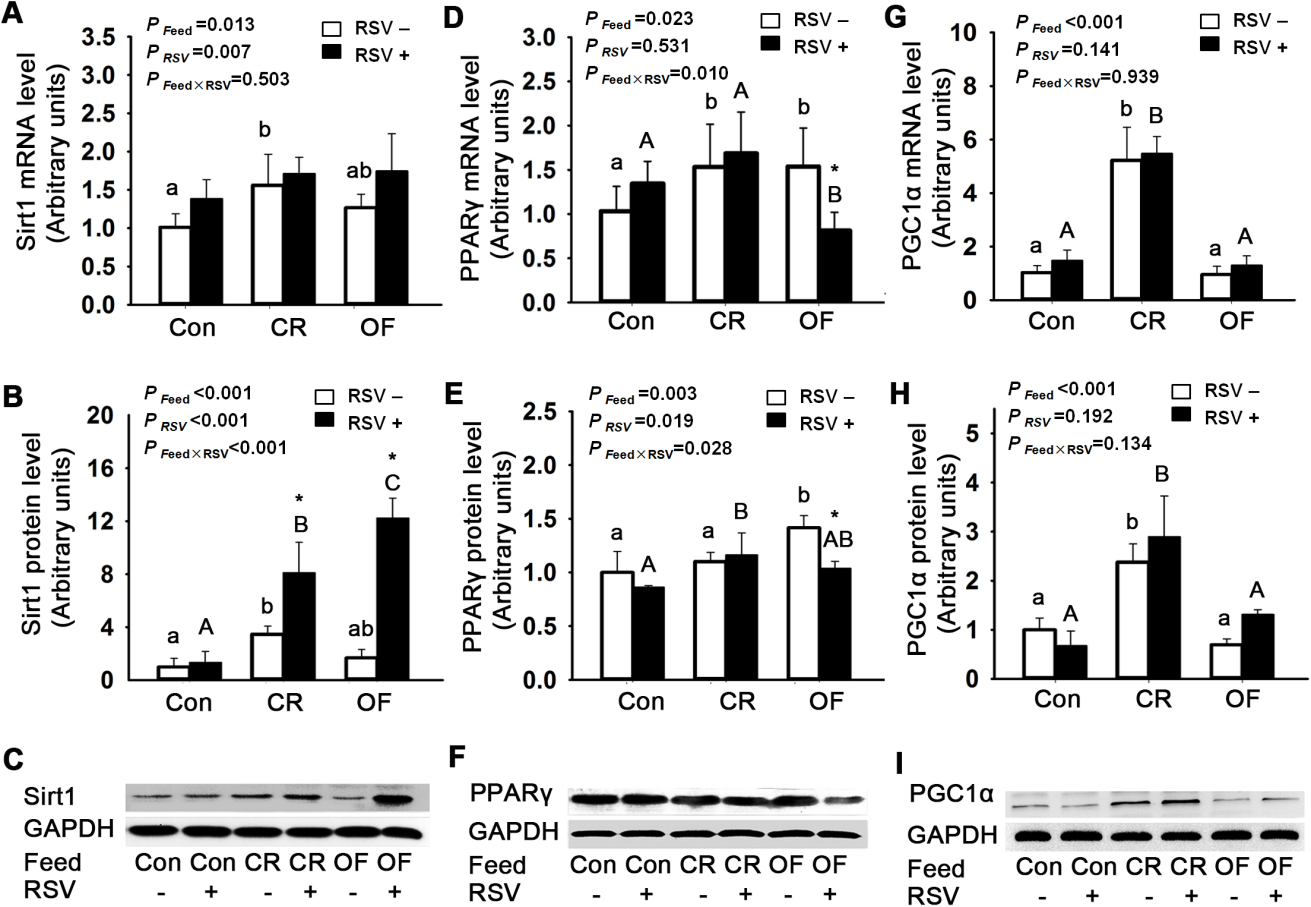
Effects of feed intake and RSV on Sirt1, PPARγ and PGC1α levels in zebrafish muscle. A. Sirt1 mRNA levels, B. Sirt1 protein levels, C. Immunoblots of Sirt1. D. PPARγ mRNA levels, E. PPARγ protein levels, F. Immunoblots of PPARγ. G. PGC1α mRNA levels, H. PGC1α protein levels, I. Immunoblots of PGC1α. The data were presented as Mean ± SD. Letters (a, b, c) indicated the multiple comparison results among various feed intake groups without RSV. Capital letters (A, B, C) indicated the multiple comparison results among various feed intake groups with RSV. Same letters indicated no significant difference, different letters indicated significant differences in statistics. * indicated the significant difference between groups without RSV and with RSV. For those there was no statistically significant difference between groups, the letters were not shown. Significance level was 0.05.

Both feed intake and RSV had significant effects on Sirt1 mRNA (*P* = 0.013) and protein levels (*P* <0.001), and their interaction effect on protein expression was also observed (*P* <0.001). Sirt1 protein level in CR group was increased by 2.5-fold (*P* <0.05) compared with Con group. RSV treatment enhanced the Sirt1 protein level in CR and OF zebrafish. Sirt1 protein level in CR+RSV group was enhanced by 1.3-fold compared to CR group, and Sirt1 protein level in OF+RSV group was increased by 6.3-fold compared to OF group (Fig 3A–3C).

Feed intake significantly affected PPARγ mRNA level (*P* = 0.023). Both feed intake and RSV had significant effects on PPARγ protein level, and their treatment effects interacted. PPARγ protein level in OF group was enhanced significantly compared with Con group and CR group (*P* <0.01). RSV treatment reduced PPARγ protein level in OF zebrafsh. PPARγ protein level in OF+RSV group was reduced by 26.9% (*P* <0.05) compared to OF group (Fig 3D–3F). Differences in feed intake significantly affected PGC1α mRNA and protein levels (*P* <0.001). PGC1α protein level of CR group was significantly enhanced compared to Con and OF groups (Fig 3G–3I).

**Fig 4:**
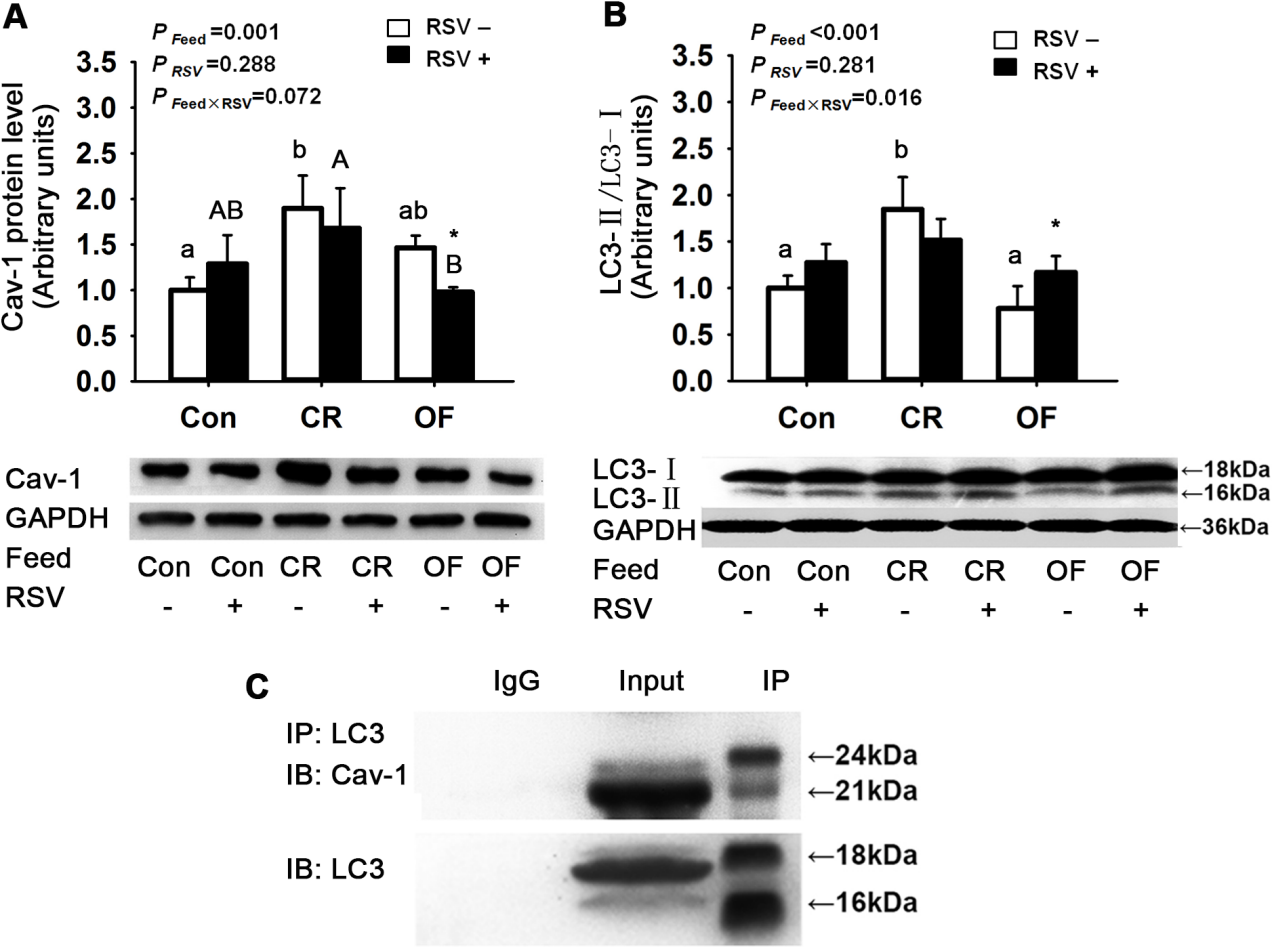
Effects of feed intake and RSV on Cav-1 and LC3 in zebrafish muscle. A. Cav-1 protein level. B. Ratio of LC3-II protein level to LC3-I protein level. C. Co-immunoprecipitation (IP) and immunoblots (IB) of Cav-1 and LC3. The data were presented as Mean ± SD. Letters (a, b, c) indicated the multiple comparison results among various feed intake groups without RSV. Capital letters (A, B, C) indicated the multiple comparison results among various feed intake groups with RSV. Same letters indicated no significant difference, different letters indicated significant differences in statistics. * indicated the significant difference between groups without RSV and with RSV. For those there was no statistically significant difference between groups, the letters were not shown. Significance level was 0.05.

Differences in feed intake affected Cav-1 protein level (*P* <0.001). Compared with Con group, Cav-1 protein level was enhanced significantly by 89.7% in CR group and increased marginally by 46.5% in OF group. Treatment with RSV significantly reduced Cav-1 protein level by 33.1% (*P* <0.01) in OF zebrafish (Fig 4A).

Differences in feed intake significantly changed LC3-II/LC3-I in zebrafish, and there was interaction effect of LC3-II/LC3-I observed between feed intake and RSV. Compared to Con group, ratios of LC3-II/LC3-I protein level in CR group were increased by 84.7% (*P* <0.001). LC3-II/LC3-I protein level was enhanced by 49.9% (*P* <0.01) in OF+RSV group compared to OF group (Fig 4B).

Association between LC3-II and Cav-1 was analyzed using co-immunoprecipitation. Results from immunoprecipitation showed that there were interactions between Cav-1 and LC3-II (Fig 4C).

## Discussion

In the present study, the effects of feed intake and RSV and the potential regulatory pathways have been investigated in zebrafish. Three levels of calorie intake, including normal (control), calorie restriction and overfeeding are applied. The energy requirement of zebrafish is 30 calories per day [26]. One mg artemia may provide 5 calories, and the consumption of artemia is about 80% [23]; therefore, eight mg cysts/fish/day is provided for control groups to meet the energy demands. For CR groups, five mg cysts/fish/day (around 60% of the control group) is provided. For overfeeding groups, 60 mg cysts/fish/day is provided referring to the literature [27].

Our results show that CR decreases but overfeeding increases body length, body weight and Fulton’s condition factor in zebrafish. The different calorie intake may be contributed to the formation of different somatotypes. It has been reported that excessive feed intake induces the development of obesity in zebrafish and certain rodent strains, and application of calorie restriction is able to reduce the increased body weight by overfeeding in zebrafish [23, 28]. The findings of the present study confirm the previous reports, suggesting the consistent effects of feed intake on body weight across species.

The dose of RSV in this study is based on a previous study, which shows that after treatment with 5 mg/L RSV (about 21.9 μmol/L), RSV is detectable in liver of zebrafish and mRNA levels of SIRT3 and SIRT4 are decreased significantly [29]. Therefore, in the present study the dose of RSV is set at 20 μmol/L.

The present study demonstrate that eight weeks of RSV treatment do not affect body length, body weight and Fulton’s condition factor in zebrafish. Previous studies show that RSV supplementation decreases body weight in overfed mice. Lagouge et al. [30] show that RSV (200 or 400 mg/kg/day) significantly attenuates weight gain and protects mice against diet-induced obesity. Furthermore, RSV treatment for 26 weeks significantly decreases body weight and high-fat diet-induced obesity in C57BL/6 mice [31]. In this study, treatment time for RSV is 8 weeks and the dose is 20 μmol/L. Reduced efficacy of RSV supplementation in the present work is probably due to the lower RSV dose, shorter treatment period, and difference in species.

The present study show that overfeeding significantly elevates blood glucose; however, treatment with RSV for 8 weeks do not attenuate blood glucose increase by overfeeding in zebrafish. To the best of our knowledge, there has been no data available on the dose-dependent effect of RSV on blood glucose. There is a potential that varying doses of RSV may exert their physiological regulation differently, which requires additional studies. Data from literature indicate the effects of RSV in decreasing blood glucose level in diet-induced rodents with hyperglycemia [32, 33]. La Fleur et al. [34] suggest that rat blood glucose could be directly affected by dietary composition. In our study, increased blood glucose in overfed zebrafish remains unaffected after RSV intake, which probably is related to differences in nutritional ingredients of fat and sugar between rodents and zebrafish. Alternatively, there could be differences between these two species in glucose metabolism and yet-to-be-identified mechanism for RSV actions.

Our results show that RSV decreases plasma TG in zebrafish on a CR diet. Additionally, treatments with RSV do decrease plasma TC levels in overfed zebrafish. It has been reported that RSV reduces circulating TG and cholesterol and prevents hepatic steatosis in overfed rodents, suggesting the effects of RSV on ameliorating lipid profiles [35, 36]. Similarly, in the present study, histological results indicate that RSV tends to ameliorate the hepatic steatosis and hepatocyte ballooning in overfed zebrafish. The present study in part confirm the ameliorating effects of RSV on lipid metabolism disregulations induced by excessive feed intake. Furthermore, the present study has attempted to identify the potential regulatory pathways of RSV for improving lipid profile and hepatic steatosis.

It has been shown that CR and RSV activates AMPKα activity, which in turn induces the expression of Sirt1 [37]. Increased expressions of phosphorylated AMPK and Sirt1 regulate lipid homeostasis and glucose metabolism, consequently preventing fat accumulation in liver and ameliorating obesity-related metabolic changes [38, 39]. Hence, in the present experiment, we evaluate protein levels of AMPKα and Sirt1 in muscle. Our findings indicate that CR substantially increases AMPKα phosphorylation and Sirt1 protein expression in zebrafish, which are in agreement with the previous studies. Moreover, the bidirectional effects of RSV on AMPKα phosphorylation is observed in zebrafish; that is, AMPKα phosphorylation in increased and decreased by RSV in overfed and CR zebrafish, respectively. Furthermore, there are interactions between feed intake and RSV, suggesting that RSV exerts different effects under different dietary stress.

Recent evidences suggest that Sirt1 could activate PGC1α *via* deacetylation with a concomitant suppression of PPARγ expression [8, 13]. Meanwhile, phosphorylated AMPKα could also repress PPARγ expression [14]. PPARγ is highly expressed in adipocytes and promotes differentiation and storage of fatty acid, it is thereby considered an ultimate effector of adipogensis [40, 41]. Down-regulation of PPARγ triggers lipolysis and loss of fat [13, 16].

In the present study, CR induces expressions of Sirt1 together with PGC1α, suggesting a potential that CR increases PGC1α protein level through activation of its upstream factor Sirt1. However, in CR zebrafish the PPARγ protein level remains unchanged, suggesting that in CR zebrafish PPARγ protein level is not regulated through the activation of Sirt1. Compared with Con group, PPARγ protein level increases in overfed zebrafish, and RSV supplementation reduces PPARγ protein expression. These results suggest that increased PPARγ protein is probably contributed to the flux of fatty acids and fat accumulation leading to the occurrence of obesity, and RSV could down-regulate PPARγ protein expression for attenuation of the disregulations of lipid metabolism under dietary stress.

Under adequate nutrition status, free fatty acids in hepatocytes are converted into TG for storage in lipid droplets. Upon nutrient deprivation, TG in lipid droplets is broken down to supply free fatty acids for oxidation to meet cellular energy requirements [42]. The role of autophagy has been a considerable concern in such pathway of lipid metabolism owing to its potential implication in obesity and metabolic syndrome. In the present study, an autophagy marker protein, LC3, is used to indicate the procession of autophagy. Our results show CR up-regulated LC3-II protein level, which suggests that autophagy is involved in the above-mentioned TG breakdown process. Such CR-induced autophagy could be effectively attenuated by RSV. Alternatively, under overfed conditions RSV may enhance LC3-II protein level, suggesting that RSV may induce the autophagy process. Histological results reveal that RSV relieves fatty infiltration in liver, suggesting that induced autophagy by RSV potentially facilitates lipid droplet breakdown and probably attenuates lipid droplet formation. Under different calorie stress, RSV exerts different effects, suggesting the regulatory roles of RSV on lipid metabolism fitness.

In the present study, CR treatments elevated LC3-II/LC3-I ratio together with Sirt1 expression. During starvation, nuclear Sirt1 becomes activated and deacetylates LC3. Then, the deacetylated LC3 re-localizes to cytoplasm through interaction with the diabetes- and obesity-regulated gene (DOR) and the LC3-DOR complex binds to autophagy-related protein 7 (Atg7). This promotes conjugation of LC3 to phosphatidylethanolamine and forms LC3-phosphatidylethanolamine conjugate (LC3-II), followed by recruitment to autophagosomal membranes and promotion of the formation of autophagosome [43]. The findings of this study suggest a potential that in zebrafish Sirt1 is involved in the initiation of autophagy.

Caveolae are specialized lipid raft structure that is formed from omega-shaped invaginations of the plasma membrane into the cytosol [44]. Cav-1 is an essential protein constituent of caveolae and has a ubiquitous function in suppression of autophagy [45]. We determine the effect of RSV on Cav-1 protein level in zebrafish under different nutritional conditions. Our results indicate CR enhances Cav-1 protein level, which is probably related to the inhibition of autophagy. Under overfed conditions, RSV down-regulates Cav-1 protein expressions, indicating the potential of RSV to induce autophagy. Additionally, under overfed conditions, RSV elevates protein expressions of LC3-II, an indicator of autophagy. The observation of down-regulation of Cav-1 together with up-regulation of LC3-II suggests RSV-induced autophagy in overfed zebrafish.

It has been reported that Cav-1 spontaneously interacts with LC3-II to promote autophagy [45] and to participate in lipid droplet formation and breakdown *in vivo* [44]. In addition, overexpression of LC3-II protein enhances its interaction with Cav-1 to form lipid rafts [36]. Thus, we evaluate the interaction effect of Cav-1 and LC3-II in an attempt to determine potential regulatory roles of Cav-1 in lipid metabolism in muscle of zebrafish. In consistence with previous reports, we also find that LC3-II directly binds Cav-1. However, specific mechanisms of Cav-1 interaction with LC3-II require additional research.

In summary, CR reduces body length, body weight, and condition factor of zebrafish. CR reduces plasma TG, but induces expressions of phosphorylated AMPKα, Sirt1, and PGC1α, indicating that CR may reduce plasma TG through activation of the AMPKα-Sirt1- PGC1α pathway. RSV suppresses AMPKα phosphorylation by CR but increases by overfeeding in zebrafish, suggesting the regulatory effect of RSV on maintenance of lipid metabolism homeostasis under different dietary stress. In overfed zebrafish, RSV increases the phosphorylation level of AMPKα, increases Sirt1 expression, and decreases the elevated protein level of PPARγ, suggesting the potential that RSV may regulate lipid metabolism through AMPKα-Sirt1-PPARγ pathway in overfed zebrafish. Additionally, RSV down-regulates Cav-1 protein levels and up-regulates LC3-II protein levels, suggesting the regulatory role of RSV in autophagy induction in OF zebrafish.

## Supporting information

S1 Table The proximate composition of artemia cysts. (XLSX)

S2 Table Effects of feed intake and RSV on AMPKα and pAMPKα protein levels in zebrafish muscle. Letters (a, b, c) indicated the multiple comparison results among various feed intake groups without RSV. Capital letters (A, B, C) indicated the multiple comparison results among various feed intake groups with RSV. Same letters indicated no significant difference, different letters indicated significant differences in statistics. * indicated the significant difference between groups without RSV and with RSV. For those there was no statistically significant difference between groups, the letters were not shown. Significance level was 0.05.
(XLSX)

S3 Table Effects of feed intake and RSV on Sirt1, PPARγ and PGC1α protein levels in zebrafish muscle. Letters (a, b, c) indicated the multiple comparison results among various feed intake groups without RSV. Capital letters (A, B, C) indicated the multiple comparison results among various feed intake groups with RSV. Same letters indicated no significant difference, different letters indicated significant differences in statistics. * indicated the significant difference between groups without RSV and with RSV. For those there was no statistically significant difference between groups, the letters were not shown. Significance level was 0.05.
(XLSX)

S4 Table Effects of feed intake and RSV on Cav-1 and LC3 protein levels in zebrafish muscle. Letters (a, b, c) indicated the multiple comparison results among various feed intake groups without RSV. Capital letters (A, B, C) indicated the multiple comparison results among various feed intake groups with RSV. Same letters indicated no significant difference, different letters indicated significant differences in statistics. * indicated the significant difference between groups without RSV and with RSV. For those there was no statistically significant difference between groups, the letters were not shown. Significance level was 0.05.
(XLSX)
